# Inflammatory back pain in psoriatic arthritis is significantly more responsive to corticosteroids compared to back pain in ankylosing spondylitis: a prospective, open-labelled, controlled pilot study

**DOI:** 10.1186/s13075-018-1565-4

**Published:** 2018-04-17

**Authors:** Muhammad Haroon, Muddassar Ahmad, Muhammad Nouman Baig, Olivia Mason, John Rice, Oliver FitzGerald

**Affiliations:** 1 Division of Rheumatology, Department of Medicine, University Hospital Kerry, Tralee, Ireland; 2 Department of Orthopaedics, University Hospital Kerry, Tralee, Ireland; 3 CSTAR (Centre for Support and Training in Analysis and Research), University College Dublin, Dublin, Ireland; 4 Department of Rheumatology, St Vincent’s University Hospital, Dublin, Ireland

**Keywords:** Corticosteroids, Psoriatic arthritis, Axial psoriatic arthritis, Ankylosing spondylitis, Inflammatory back pain

## Abstract

**Background:**

The efficacy of corticosteroids in patients with psoriatic arthritis (PsA) and inflammatory back pain has not been studied to date. In this controlled trial, we aimed to investigate the comparative performance of corticosteroids in patients with active axial-PsA (AxPsA) versus those with active ankylosing spondylitis (AS).

**Methods:**

Patients with AxPsA and AS (naïve to biologic therapies), who not only had clinically active disease, but also had bone marrow oedema on magnetic resonance imaging of the sacroiliac joints, were recruited. Clinically active disease was defined as inflammatory back pain (fulfilling Assessment of Spondyloarthritis International Society (ASAS) expert criteria), with spinal pain score (numerical rating scale 0–10) ≥4 and Bath AS Disease Activity Index (BASDAI) score ≥4 despite taking nonsteroidal anti-inflammatory drugs. Moreover, we recruited a control group of patients with non-inflammatory lower back pain. All patients received a single, intra-muscular dose of depot corticosteroid injection (triamcinolone acetonide 80 mg) at baseline. The intra-muscular corticosteroid option was used to overcome any drug compliance issues. Clinical outcome assessments were made at the following time points: baseline, week 2, and week 4. The primary efficacy end point was mean change in Ankylosing Spondylitis Disease Activity Score (ASDAS) at week 2. Key secondary outcomes were mean change in the BASDAI, Bath Ankylosing Spondylitis Functional Index (BASFI) and Ankylosing Spondylitis Quality of Life (ASQoL) at weeks 2 and 4.

**Results:**

In total, 40 patients were recruited (15 with AxPsA, 15 with AS, and 10 controls). At week 2 following corticosteroid treatment, patients with AxPsA had significantly greater improvement in the mean ASDAS compared to patients with AS (1.43 ± 0.39 vs. 1.03 ± 0.30, *p* = 0.004), and also when compared to controls (*p* < 0.001). At week-4, similar significant trend of ASDAS improvement was seen among AxPsA patients compared to AS patients (1.09 ± 0.32 vs. 0.77 ± 0.27, *p* = 0.007) and controls (*p* < 0.001). Similarly, the mean BASDAI, visual analogue scale spinal pain score, ASQoL and BASFI improved significantly among patients with AxPsA compared to patients with AS and controls at week 2 (*p* < 0.05), with this trend also largely maintained at week 4.

**Conclusions:**

Axial inflammation in patients with PsA responds significantly better to corticosteroids than in patients with AS. This furthers the argument and adds to the growing evidence that AxPsA and AS are distinct entities.

## Background

Psoriatic arthritis (PsA) is a progressive, immune-mediated musculoskeletal disease with involvement of synovial, entheseal and axial structures. There are varied reports of its prevalence among patients with psoriasis (PsO), and it is becoming clear that PsA is much more common than previously thought. We have shown that 29% of patients with PsO attending dermatology clinics had undiagnosed PsA [1]. PsA was formerly considered a milder form of arthritis but an inception cohort study has shown that 47% of the patients with PsA who presented within 5 months of onset of symptoms had ≥1 erosion by the second year of follow up, despite the fact that the majority had been treated with disease-modifying anti-rheumatic drugs (DMARDs) [2]. More recently, we have also shown that even a 6-month delay from symptom onset to the first visit with a rheumatologist can lead to the development of peripheral joint erosions and worse long-term physical function [3].

It is common that patients with PsA with peripheral arthritis have concomitant inflammatory axial disease, but isolated inflammatory axial disease occurs in fewer than 5% of patients [4]. The reported prevalence of axial disease in patients with PsA is quite variable, and has been reported to be as high as up to 78% [5]. At present there are no approved therapies for treatment of axial involvement in PsA. Most of the treatment response data about axial involvement in SpA come from ankylosing spondylitis (AS) studies, while data about the response of axial involvement in PsA to various treatment strategies are limited [6]. It remains to be seen whether the availability of new therapeutic agents (such as IL-17 inhibitors and IL-23 inhibitors) will have any beneficial effect in patients with axial PsA (AxPsA). The efficacy of corticosteroids in patients with PsA and inflammatory back pain (IBP) has not been studied to date.

AS is a chronic inflammatory disorder of unknown aetiology that principally involves the axial joints and adjacent structures leading to progressive bony fusion of the spine. Nonsteroidal anti-inflammatory drugs (NSAIDs) and biologic drugs such as TNF inhibitors (TNFi) are the principle treatments. Although very little is known about the use of corticosteroids in AS, a recent double-blind, randomised, placebo-controlled trial has shown that 50 mg of prednisolone was only partially effective in patients with active AS, while a dose of 20 mg was not effective [7].

A number of studies have suggested that there are clinical, radiologic, and genetic differences between axial disease in PsA (AxPsA) and AS, suggesting that these are distinct entities [8–10]. Similarly, the studies examining typical AS-associated genetic risks in AxPsA have largely been negative, further supporting the theory that spinal involvement in PsA is genetically different from that seen in AS [11]. However, no study to date has directly compared the therapeutic response in these two diseases. There is only one study that looked at the response of TNFi in active AS (fulfilling modified 1984 New York Criteria for AS) with and without concomitant psoriasis (but no formal diagnosis of PsA) [12]. In this controlled trial, we aimed to investigate the comparative performance of corticosteroids in patients with active axial-PsA versus those with active AS.

## Method

All patients attending rheumatology clinics at University Hospital Kerry, who had a confirmed diagnosis of PsA as per the internationally agreed Criteria of the Classification of Psoriatic Arthritis (CASPAR) [13], and AS as per the 1984 Modified New York criteria [14], were suitable for inclusion. Among them, patients with active AxPsA and active AS were recruited in this open-label controlled trial. Active disease was defined as IBP (fulfilling Assessment of Spondyloarthritis International Society (ASAS) expert criteria) [15], with a spinal pain score (numerical rating scale 0–10) ≥4 and Bath Ankylosing Spondylitis Disease Activity Index (BASDAI) score ≥4 despite taking NSAIDS. Furthermore, only those patients with AxPsA and AS with magnetic resonance imaging (MRI)-proven sacroiliac (SI) joint bone marrow oedema [16] (MRI of the sacroiliac joints performed within the 6 months prior to recruitment) were considered for inclusion. Hence, all recruited patients with AxPsA and AS had not only clinically active disease, but also had bone marrow oedema on MRI of the SI joints. Radiographs and MRI scans were reviewed by a consultant radiologist. We further scored inflammation by MRI in the SI joints using the Spondyloarthritis Research Consortium of Canada Magnetic Resonance Imaging Index [17].

All recruited patients were naive to biological therapies such as anti-TNF alpha agents. Moreover, we recruited a control group of patients with non-inflammatory lower back pain attending orthopaedic clinics. Patients in this control group suffered from discogenic lower back pain, and were matched by age, gender and severity of back pain with patients with AxPsA and AS. Patients excluded were those under the age of 18 years and patients with learning difficulties, peripheral joint active polyarthritis, or concomitant fibromyalgia. Patients with monoarthritis or oligoarthritis were included in the study. All patients received a single, intra-muscular dose of depot corticosteroid injection (triamcinolone acetonide 80 mg) at baseline. The intra-muscular corticosteroid option was used to overcome any drug compliance issues. Clinical outcome assessments were made at following time points: baseline, week 2, and week 4. Assessments included the following: spinal pain score (numerical rating scale 0–10), BASDAI, Bath AS Functional Index (BASFI), Ankylosing Spondylitis Quality of Life (ASQoL), Ankylosing Spondylitis Disease Activity Score (ASDAS), patient global assessment (numeric rating scales 0–10), number of swollen joints (66/68-joint score) and number of tender entheseal sites (Leeds Enthesitis Index). Laboratory outcome assessments included measurement of C-reactive protein (CRP). The primary efficacy end point was the mean change in ASDAS at week 2. Key secondary outcomes were mean change in BASDAI, BASFI, and ASQoL at week 2 and week 4.

ASDAS is a validated composite index that combines patient-oriented measures with a laboratory measure of inflammation (CRP level or erythrocyte sedimentation rate (ESR)) [18], and is used to monitor the actual level of disease activity and to measure response to treatment. As per the recommendations of the Assessment of Spondyloarthritis International Society (ASAS), ASDAS values of 2.1 and 3.5 were selected as cutoff values to define high and very high disease activity, respectively, and ASDAS <1.3 was used as a cutoff to define inactive disease [18]. Similarly, the cutoffs for improvement scores were a change ≥1.1 units for “clinically important improvement” and a change ≥2.0 units for “major improvement” [19].

The study was approved by the local Medical Research Ethics committee (Clinical Research Ethics Committee of the Cork Teaching Hospitals, University College Cork, Ireland; reference number ECM 4, 19/1/16). Written informed consent was obtained from all participants. There were no missing data, as patients were assessed in a dedicated research clinic where all aforementioned clinical, laboratory, and radiographic details were collected.

The required sample size was estimated using the software G*Power 3.1.9.2. With β of 0.8, α of 0.05 and effect size *f* of 0.25, the total sample size calculated was 30, which reflects actual power of 84%. Statistical analysis was performed using the SPSS software, version 21. Significance was defined as *p* < 0.05 (two-tailed). Baseline descriptive statistics were computed with continuous variables summarised by their means and SD; categorical variables were summarised by proportions. The chi square (X2) statistic was used to investigate the distributions of categorical variables, and continuous variables were analysed using Student’s *t* test.

## Results

In total, 40 patients were recruited (15 with AxPsA, 15 with AS, and 10 controls). Table 1 provides the baseline demographic characteristics of these patients, showing that these groups were well-matched by age, gender, and severity of back pain. Sixty percent of the patients with AxPsA were using disease-modifying anti-rheumatic drugs (DMARDs) for their peripheral joint disease. Amongst the AxPsA patients, the mean swollen joint count, tender joint count and Leeds Enthesitis Index was 1.8, 2.7, and 0.2, respectively. The Spondyloarthritis Research Consortium of Canada (SPARCC) score in the AS and AxPsA groups was 24.60 ± 4.68 and 21.86 ± 2.53, respectively.

**Table 1:** Baseline demographic characteristics and clinical parameters among three patient groups – the axial psoriatic arthritis, ankylosing spondylitis, and control groups

Parameter	Axial psoriatic arthritis	Ankylosing spondylitis	Control
Number of patients	15	15	10
Age in years (mean ± SD)	38.6 ± 7.93	33.9 ± 8.28	40.0 ± 11.6
Duration of back pain - years (mean ± SD)	4.5 ± 1.3	5.4 ± 1.4	5.6 ± 1.7
Gender (female %)	60	46.7	60
CRP (mg/L)	9.00 ± 4.74	7.27 ± 3.61	4.00 ± 1.56
VAS (mean ± SD)	6.60 ± 1.12	6.47 ± 1.13	6.60 ± 1.51
ASQOL (mean ± SD)	11.8 ± 4.19	11.9 ± 3.74	10.0 ± 1.49
BASFI (mean ± SD)	6.40 ± 1.52	6.06 ± 1.49	4.14 ± 1.53
BASDAI (mean ± SD)	5.95 ± 1.24	5.86 ± 1.32	5.18 ± 1.07
ASDAS (mean ± SD)	3.81 ± 0.61	3.75 ± 0.55	2.82 ± 0.67

There were no significant differences (*p* > 0.05) in the parameters listed in Table 1 between patients with axial psoriatic arthritis (AxPsA) and patients with ankylosing spondylitis (AS). Similarly, there were no significant differences (*p* > 0.05) in age, gender, visual analogue scale (VAS) spinal pain score, Ankylosing Spondylitis Quality of Life (ASQoL) or Bath Ankylosing Spondylitis Disease Activity Index (BASDAI) in patients with AxPsA or AS compared with controls

*CRP* C-reactive protein, *BASFI* Bath Ankylosing Spondylitis Functional Index, *ASDAS* Ankylosing Spondylitis Disease Activity Score

**Fig. 1:**
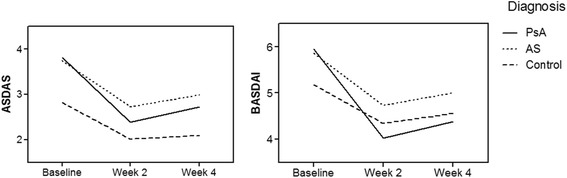
Changes in mean ankylosing Spondylitis Disease Activity Score (ASDAS) and Bath Ankylosing Spondylitis Disease Activity Index (BASDAI) over time by diagnosis. PsA, psoriatic arthitis; AS, ankylosing spondylitis

**Fig. 2:**
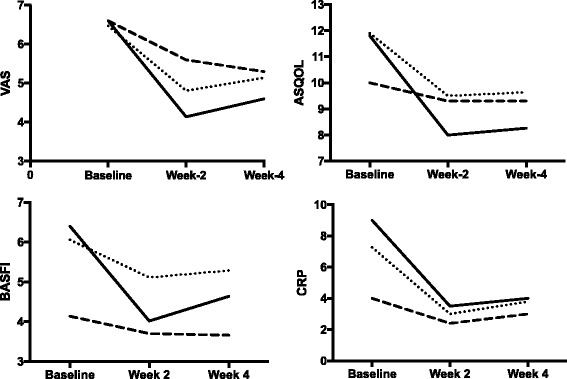
Changes in mean visual analogue scale (VAS) spinal pain score, Ankylosing Spondylitis Quality of Life (ASQOL), Bath Ankylosing Spondylitis Functional Index (BASFI), and C-reactive protein (CRP) over time by diagnosis. The diagnostic categories are represented similar to Fig. 1

At week 2 following corticosteroid treatment, patients with AxPsA had significantly greater improvement in the mean ASDAS compared to patients with AS (1.43 ± 0.39 vs. 1.03 ± 0.30, *p* = 0.004), and the same was the case when compared to controls (*p* < 0.001, Table 2, Fig. 1). At week 4, patients with AxPsA also had significantly greater improvement in the mean ASDAS compared to both patients with AS (1.09 ± 0.32 vs. 0.77 ± 0.27, *p* = 0.007) and controls (*p* < 0.001). Similarly, the mean BASDAI, VAS spinal pain score, ASQoL and BASFI improved significantly among patients with AxPsA compared to patients with AS and controls at week 2, with this trend also largely maintained at week 4 (Table 2, Figs. 1 and 2). Figure 1 shows the mean changes in ASDAS and BASDAI, and Fig. 2 shows the mean changes in VAS spinal pain score, BASFI, ASQOL, and CRP at baseline, week 2 and week 4 among patients with AxPsA and AS and controls.

**Table 2:** Primary and secondary outcome measure responses at week 2 and week 4 of corticosteroid treatment

Parameter	Axial psoriatic arthritis	Ankylosing spondylitis	Control	*P* value, AxPsA vs. AS
Mean difference from baseline to week 2				
ASDAS	1.43 ± 0.39	1.03 ± 0.30	0.81 ± 0.26	0.004
VAS	2.46 ± 0.91	1.66 ± 1.1	1.0 ± 0.94	0.003
ASQoL	3.80 ± 1.82	2.4 ± 1.72	0.7 ± 0.67	<0.001
BASFI	2.38 ± 0.68	0.95 ± 0.91	0.44 ± 0.41	<0.001
BASDAI	1.93 ± 0.56	1.13 ± 0.33	0.84 ± 0.24	<0.001
Mean difference from baseline to week 4				
ASDAS	1.09 ± 0.32	0.77 ± 0.27	0.73 ± 0.24	0.007
VAS	2.00 ± 0.92	1.33 ± 0.72	1.30 ± 0.82	0.054
ASQoL	3.53 ± 1.35	2.26 ± 1.53	0.70 ± 0.67	<0.001
BASFI	1.76 ± 0.82	0.78 ± 0.63	0.48 ± 0.24	<0.001
BASDAI	1.57 ± 0.49	0.85 ± 0.45	0.62 ± 0.23	<0.001

Data are presented as (mean ± SD)

AxPsA axial psoriatic arthritis, AS ankylosing spondylits, *VAS* visual analogue scale, *ASQoL* Ankylosing Spondylitis Quality of Life, *BASDAI* Bath Ankylosing Spondylitis Disease Activity Index, *BASFI* Bath Ankylosing Spondylitis Functional Index, *ASDAS* Ankylosing Spondylitis Disease Activity Score

Additionally, we compared the mean ASDAS in patients with AS and controls, and found that at week 2 of corticosteroid challenge there was marginal improvement in ASDAS (1.02 ± 0.30 vs. 0.81 ± 0.25, *p* = 0.077), but at week 4 there were no significant mean improvement in ASDAS (0.76 ± 0.27 vs.0.74 ± 0.22, *p* = 0.79). However, there was significantly greater improvement in BASDAI among patients with AS compared to controls at week 2 (1.12 ± 0.33 vs. 0.84 ± 0.24, *p* = 0.02), but at week 4 there was no significant improvement in mean BASDAI (0.85 ± 0.45 vs. 0.62 ± 0.23, *p* = 0.15).

## Discussion

Inflammatory spinal disease is one of three inflammatory musculoskeletal manifestations that frequently occur in PsA. There are very limited data about the axial involvement in PsA, especially as regards treatment, with treatment guidelines based largely on data from AS trials. To our knowledge, this is the first study to specifically investigate axial disease in PsA. Since at least 50% of patients with PsA develop inflammatory axial disease and it has been shown that limitation of spinal mobility and spinal radiologic changes increase over time, it is important to examine the commonly available therapeutic options [20, 21]. This is especially important as there remains a question as to whether the inflammatory axial disease in PsA may be similar or different to that described in Axial SpA.

The results of our study are important in a number of ways. First, we have carefully compared the efficacy of corticosteroids among patients with AxPsA and AS with IBP and a control group of patients with non-inflammatory lower back pain. This study shows very clearly that patients with AxPsA respond significantly better to corticosteroids than both patients with AS and controls. This observation was quite consistent across the different clinical parameters such as ASDAS, spinal pain score (measured by VAS), BASFI, ASQoL, and BASDAI, and was recorded at both week 2 and week 4. Some attenuation of clinical responses at week 4 compared to week 2 is not surprising and is consistent with gradual wearing-off of the corticosteroid effect over time. Clearly, in our study ASDAS improved significantly in patients with AxPsA both at week 2 and week 4. Furthermore, if we examine the ASDAS improvement criteria, only patients with AxPsA achieved “clinically important improvement” status at week 2. At week 4, there was mild attenuation of the response with the mean change in ASDAS of 1.09 (mean change of 1.1 is required for clinical important improvement) [19].

Second, the significant improvement in IBP in patients with AxPsA compared to those with AS suggests that IBP may have a different pathogenesis in AxPsA and that different therapies might be considered. This is the first study to date comparing the therapeutic response in these two diseases. It is worth noting that the patients with AxPsA in our study responded significantly better to corticosteroids, although they had a relatively lesser burden of bone marrow oedema than the patients with AS: however, patients with AxPsA had other comparable features of back pain. One plausible explanation is that there was more inflammatory involvement in the spine than in the SI joints in these patients with AxPsA, as it has been shown that spinal changes may develop in the absence of SI involvement [22, 23]. Moreover, among patients with AxSpA, spinal inflammation has been observed on MRI in up to half of those without MRI evidence of SI joint inflammation [24, 25]. Unfortunately, we do not have MRI of spine on our patients to further investigate this concept. Another plausible explanation lies in the hypothesis that in PsA there are different primary sites of inflammation, such as synovial-predominant, or enthesis-predominant [26], and this can potentially differentiate patients with AxPsA from those with AS. One can speculate that the IBP in AxPsA is predominantly synovial-based*.* Interestingly, it has been shown that synovial-based phenotypes in PsA such as joint deformities and joint fusion are associated with HLA-B*08, and similarly, sacroiliitis in PsA is most commonly associated with HLA-B*08 [27]. This is supported by previous studies showing that axial disease in PsA is significantly associated with severe peripheral joint disease [28, 29]. Since PsA is a heterogeneous disease, probably the axial involvement in most of AxPsA patients is predominantly synovial-based and associated with HLA-B*08, and in others, entheseal-based and associated with HLA-B*27 [27]. Given the marked improvement in IBP in AxPsA with corticosteroids, we would propose that standard synthetic (s)DMARDs should be formally tested in active AxPsA, as responses may also be different to those seen in AS where they have not shown benefit.

The strengths of our study include the following: (1) the inclusion of a control group with non-inflammatory lower back pain, and (2) the use of a more robust approach in defining active AxPsA for inclusion of patients in this study - the combination of both active IBP along with the presence of MRI-proven SI joint bone marrow oedema. The rationale to use this approach was the possible disconnect between symptoms of IBP and the imaging diagnosis of sacroiliitis. For example, in a previous study of patients with psoriasis and spondylitic lesions on radiographs, IBP was reported in only 19% [30]. Furthermore, about one third of patients with PsA have asymptomatic sacroiliitis on imaging [31, 32]. We acknowledge that the small number of patients in this study is certainly a limitation of this pilot study; however, given the convincing differential response of IBP to corticosteroid treatment, the study provides useful information worthy of testing in larger and more long-term prospective studies. Genotyping data were not collected, and given such a small sample size this information might not be very helpful. Furthermore, this is an observational, non-randomised study that did not include a placebo-control group. Therefore, the results should be considered as hypothesis-generating rather than proof of efficacy of this treatment. One can argue that patients with AxPsA in our study might have more peripheral arthritis, which usually responds well to corticosteroid therapy, and this potentially could have influenced the axial symptoms along with an impact on measures of axial disease, in particular BASDAI. Measures of joint and entheseal involvement were low in our AxPsA patients with a mean swollen joint count of 1.8, tender joint count of 2.7 and Leeds Enthesitis Index of 0.2. Furthermore, we have analysed the individual questions within the BASDAI questionnaire in our patients, and note that for the questions relating to peripheral arthralgia/swelling and enthesis (question 3 and 4) the average baseline scores were only 2 and 3, suggesting that these peripheral features had little impact on BASDAI scores.

## Conclusions

We conclude that axial inflammation in PsA potentially responds significantly better to corticosteroids than in patients with AS. This furthers the argument and adds to the growing evidence that AxPsA and AS are distinct entities. Future studies should further investigate the use of corticosteroids and of sDMARD usage among patients with active IBP in PsA.

## Abbreviations

AS: Ankylosing spondylitis
ASAS: Assessment of Spondyloarthritis International Society
ASDAS: Ankylosing Spondylitis Disease Activity Score
ASQoL: Ankylosing Spondylitis Quality of Life
AxPsA: Axial psoriatic arthritis
BASDAI: Bath Ankylosing Spondylitis Disease Activity Index
BASFI: Bath Ankylosing Spondylitis Functional Index
CASPAR: Criteria of the Classification of Psoriatic Arthritis
DMARDs: Disease-modifying anti-rheumatic drugs
IBP: Inflammatory back pain
MRI: Magnetic resonance imaging
NSAIDs: Nonsteroidal anti-inflammatory drugs
PsA: Psoriatic arthritis
PsO: Psoriasis
TNFI: Tumor necrosis factor inhibitors
sDMARDs: synthetic disease-modifying anti-rheumatic drugs
SI: Sacroiliac
VAS: Visual analogue scale
